# Do Dopaminergic Impairments Underlie Physical Inactivity in People with Obesity?

**DOI:** 10.3389/fnhum.2016.00514

**Published:** 2016-10-14

**Authors:** Alexxai V. Kravitz, Timothy J. O'Neal, Danielle M. Friend

**Affiliations:** ^1^National Institutes of Health, National Institute of Diabetes and Digestive and Kidney DiseasesBethesda, MD, USA; ^2^National Institutes of Health, National Institute on Drug AbuseBaltimore, MD, USA

**Keywords:** obesity, dopamine, exercise, physical activity, physical activity promotion, Parkinson's disease, movement disorders

## Abstract

Obesity is associated with physical inactivity, which exacerbates the negative health consequences of obesity. Despite a wide consensus that people with obesity *should* exercise more, there are few effective methods for increasing physical activity in people with obesity. This lack is reflected in our limited understanding of the cellular and molecular causes of physical inactivity in obesity. We hypothesize that impairments in dopamine signaling contribute to physical inactivity in people with obesity, as in classic movement disorders such as Parkinson's disease. Here, we review two lines of evidence supporting this hypothesis: (1) chronic exposure to obesogenic diets has been linked to impairments in dopamine synthesis, release, and receptor function, particularly in the striatum, and (2) striatal dopamine is necessary for the proper control of movement. Identifying the biological determinants of physical inactivity may lead to more effective strategies for increasing physical activity in people with obesity, as well as improve our understanding of why it is difficult for people with obesity to alter their levels of physical activity.

## Introduction

Obesity is associated with reductions in motor output, often termed “physical inactivity” (Tudor-Locke et al., 2010; Bouchard et al., 2015), although whether this relationship is causal remains a point of debate (Simon et al., 2008; Haskell et al., 2009; Dwyer-Lindgren et al., 2013; Swift et al., 2014). Despite the importance of physical activity for health, there are few effective methods for increasing physical activity levels in people with obesity, leading some researchers to conclude that, “there are presently no evidence-based interventions that can reliably and sustainably increase the level of physical activity among obese adults” (Ekkekakis et al., 2016). This point is reflected in our limited understanding of the cellular and molecular determinants of physical inactivity in people with obesity. We believe that a cellular understanding of *why* obesity is associated with physical inactivity is needed to understand, and ultimately alter, the relationship between obesity and physical inactivity. In this review, we propose that impairments in striatal dopamine contribute to physical inactivity in obesity, akin to classic movement disorders such as Parkinson's disease.

**Figure 1:**
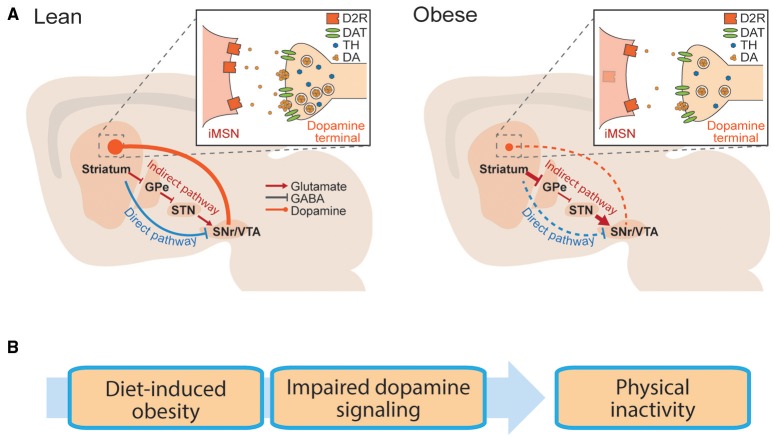
**Basal ganglia circuitry in lean and obese conditions**. **(A)** Striatal neurons send projections to the midbrain via the direct pathway or indirect pathway. Schematic is replicated in lean (left) and obese (right) conditions, to show reported dopaminergic alterations in obesity. Inlay: Dopaminergic synapse onto striatal iMSNs. GPe, globus pallidus; STN, subthalamic nucleus; SNr, substantia nigra; VTA, ventral tegmental area. **(B)** Hypothetical progression by which diet induced obesity is associated with impaired striatal dopamine transmission, leading to physical inactivity.

The striatum is a forebrain structure that controls movement, as well as learning and emotional states. There are two main projection cell types in the striatum, the “direct” and the “indirect” pathway medium spiny neurons (dMSNs and iMSNs), as well as several classes of interneurons. dMSNs and iMSNs exhibit distinct protein expression patterns, projection targets, and support distinct behavioral functions (Alexander and Crutcher, 1990; DeLong, 1990; Gerfen et al., 1990; Graybiel et al., 1994; Le Moine and Bloch, 1995; Obeso et al., 2000; Figure 1A). dMSNs express the excitatory G_s_-coupled dopamine D_1_ receptor (D1R), while iMSNs express the inhibitory G_i_-coupled dopamine D_2_ receptor (D2R; Gerfen et al., 1990). Dopamine can facilitate movement by binding to D1Rs and enhancing the output of dMSNs, or binding to D2Rs and inhibiting the output of iMSNs (Sano et al., 2003; Buch et al., 2005; Durieux et al., 2009; Kravitz et al., 2010). In this way, dopaminergic signaling controls the downstream signaling of dMSNs and iMSNs, and resulting motor output. We have simplified this discussion for the purposes of this review, but striatal function is also influenced by several additional layers of complexity (Mink, 1996; Calabresi et al., 2014). For example, the dorsal striatum is commonly linked to motor control, while the ventral striatum is linked to motivation and effortful movement (Mogenson et al., 1980; Voorn et al., 2004; Kreitzer and Malenka, 2008).

The importance of dopamine for proper control of movement is evident in neurological disorders. Hypokinetic states such as Parkinson's disease are the result of too little striatal dopamine (Hornykiewicz, 2010), whereas hyperactive states such as bipolar mania are associated with too much (Logan and McClung, 2016). Drugs that increase dopamine release (e.g., amphetamine) increase motor output (Schindler and Carmona, 2002) and dopamine antagonists (used clinically to reduce manic episodes) often result in motor impairments as a side effect (Janno et al., 2004; Parksepp et al., 2016). Genetic manipulations in animals further support the role of striatal dopamine transmission in motor control, as mice lacking dopamine receptors have reduced movement (Drago et al., 1994; Xu et al., 1994; Baik et al., 1995; Kelly et al., 1997; Beeler et al., 2016), whereas those that overexpress dopamine receptors are hyperactive (Ikari et al., 1995; Ingram et al., 1998; Dracheva et al., 1999; Thanos et al., 2001; Trifilieff et al., 2013). In particular, cell-type specific reductions of the D2R in iMSNs reduce open field movement, demonstrating the sufficiency of the D2R to regulate physical activity, by controlling the output of iMSNs (Anzalone et al., 2012; Lemos et al., 2016). In summary, striatal dopamine promotes movement in animals, due to actions on its striatal target neurons.

Obesity is associated with impairments in striatal dopamine function. Reported impairments include deficiencies in dopamine synthesis and release, as well as alterations in striatal dopamine receptors. While alterations in striatal DA transmission are commonly discussed in relation to reward processing (Kenny et al., 2013; Volkow et al., 2013), we hypothesize that these impairments may also contribute to the link between obesity and physical inactivity (Figure 1B).

## Obesity and physical inactivity

An inverse relationship between weight gain and physical activity has been observed in humans (Hemmingsson and Ekelund, 2007; Chaput et al., 2012; Hjorth et al., 2014), non-human primates (Wolden-Hanson et al., 1993), domesticated animals (Morrison et al., 2013), and rodents (Jürgens et al., 2006; Bjursell et al., 2008). The cross-species nature of this relationship indicates that it is a conserved phenomenon that may stem from the evolutionary benefit of storing energy in times of caloric excess, a state that is rare in nature. However, in modern environments physical inactivity exacerbates the negative health effects of obesity, increasing the risk of cardiac disease and diabetes (Al Tunaiji et al., 2014; Bao et al., 2014; Bouchard et al., 2015). It is possible that physical inactivity precedes, and thereby contributes to, weight gain (Jürgens et al., 2006; Haskell et al., 2009). Indeed animals with high levels of spontaneous physical activity are partially protected against diet-induced obesity (Teske et al., 2012; Zhang et al., 2012). While pre-existing differences in activity levels may contribute to the relationship between obesity and physical inactivity, at a cellular level it remains unclear *why* people with obesity are inactive.

Part of the difficulty in understanding this relationship stems from the multifaceted nature of the two variables. For instance, the weight of excess adiposity restricts joint and muscle mobility and increases joint pain, which may make it more difficult for people to move (Belczak et al., 2014; Muramoto et al., 2014). However, weight alone does not appear sufficient to explain physical inactivity in people with obesity. Several researchers have tracked physical activity levels across periods of weight loss, to see whether physical activity levels increase as people lose weight, and experience fewer mobility-restricting effects of excess adiposity. Surprisingly, weight loss is generally associated with *decreases*, and not increases, in physical activity (de Boer et al., 1986; de Groot et al., 1989; Martin et al., 2007; Redman et al., 2009). These results have been explained in terms of metabolic adaptations, as the body seeks to reduce energy expenditure to compensate for the caloric deficit induced by the diet. However, when subjects were tracked during maintained periods of weight loss lasting a year, physical activity levels still did not increase above pre-diet obese levels (Camps et al., 2013). Similar results have been reported following gastric bypass surgery. Despite large amounts of weight loss (>30 kg), objectively measured physical activity levels did not increase in patients that received gastric bypass surgery, even up to 12 months after the peak of the weight loss (Bond et al., 2010; Ramirez-Marrero et al., 2014; Berglind et al., 2015, 2016). Studies in animals also support these conclusions, as loss of adiposity is again associated with decreases, and not increases, in physical activity (Sullivan and Cameron, 2010; Morrison et al., 2014; Vitger et al., 2016). We conclude that the weight of excess adiposity does not sufficiently explain the association between obesity and physical inactivity. Rather, the evidence suggests that obesity-induced adaptations continue to contribute to physical inactivity, even after weight loss. While these adaptations may include chronic mobility issues in joints or muscles, we hypothesize that motor circuitry in the brain is also a large contributor. Specifically, we hypothesize that deficits in striatal dopaminergic signaling contribute to the persistent reductions in physical activity in obesity.

Further supporting the conclusion that the weight of adiposity does not adequately explain physical inactivity in obesity, not all groups of obese animals, or people with obesity, have low levels of physical activity. Even in studies that report deficits in striatal dopamine, physical activity levels can remain unaltered (Davis et al., 2008). Similar findings have been reported under controlled conditions in humans as well. In an 8-week study in which subjects were over-fed by 1000 calories per day, subjects significantly increased their spontaneous physical activity, despite gaining an average of 4.7 kg. The authors linked this increase to a mechanism for dissipating excess energy to preserve body weight (Levine et al., 1999). A similar increase in physical activity was reported in an 8-week over-eating study, despite an average weight gain of 5.3 kg (Apolzan et al., 2014). While physical inactivity is a correlate of obesity in large populations, there is considerable variability on this point among individuals. This variability may be another avenue for unraveling the cellular underpinnings of the relationship between physical activity and obesity.

## Obesity and disruptions in dopamine production and release

A wealth of animal research has described alterations in the dopamine system in obesity. The majority of studies in obese rodents have focused on dopamine transmission in the nucleus accumbens (NAc), which resides in the ventral striatum and is involved in effortful movement (Salamone et al., 2007; Schmidt et al., 2012). Based on this role, the NAc may be particularly important for explaining the lack of vigorous physical activity in obesity (Ekkekakis et al., 2016). Long-term *ad libitum* high-fat diet decreased tonic dopamine in the NAc of mice (Carlin et al., 2013) as well as dopamine turnover in the NAc of rats (Davis et al., 2008). This specific deficit was distinct from adiposity, as rats that were fed an iso-caloric amount of high-fat diet also had decreased dopamine turnover (Davis et al., 2008). Whereas both chow and high-fat diet increased phasic dopamine in the NAc of lean rats, obese rats had a blunted response to these diets (Geiger et al., 2009). Chronic exposure may be necessary for deficits in phasic dopamine signaling, as they are seen following 6, but not 2, weeks of high-fat diet (Cone et al., 2013). Similar to differences observed in phasic dopamine release in the NAc of obese animals, rats that were bred to be prone to weight gain had reduced dopaminergic responses to both chow (Geiger et al., 2008) and high-fat diet (Rada et al., 2010).

The above deficits in dopamine release may be explained by alterations in genes involved in the synthesis and metabolism of dopamine. Midbrain dopamine regions including the substantia nigra and the ventral tegmental area (VTA) provide the main dopaminergic innervation to the striatum (Figure 1). Expression of tyrosine hydroxylase, the rate-limiting enzyme in dopamine synthesis, is reduced in the VTA of mice fed a high-fat diet (Vucetic et al., 2012; Carlin et al., 2013). Again, this did not depend on fat storage, as similar effects were observed in mice that were pair-fed a high fat diet (Li et al., 2009). The effect of high-fat diet on co-acetyl methyl transferase (COMT), a key enzyme responsible for the degradation of dopamine is less clear, with studies reporting either decreased (Carlin et al., 2013) or unchanged (Alsio et al., 2010; Vucetic et al., 2012) expression following diet-induced obesity. Interestingly, in humans, polymorphisms that confer low activity of monoamine-oxidases (the other main enzyme responsible for degrading dopamine) have been linked to obesity (Camarena et al., 2004; Ducci et al., 2006; Need et al., 2006). Overall, the evidence supports two conclusions: (1) exposure to high-fat diets can impair dopamine synthesis and striatal dopamine release and processing, but (2) heterogeneity exists among these reports, indicating that the impact of high-fat diets on the dopamine system is complex and may occur differently among different individuals.

## Obesity and dysfunction of dopamine receptors

Multiple researchers have observed alterations in dopamine receptors in people with obesity. Individuals with at least one copy of the *drd2* Taq1A allele have reduced brain D2R availability of ~30–40% (Noble et al., 1991; Thompson et al., 1997) and an increased prevalence of obesity (Blum et al., 1996; Stice et al., 2008, 2010; Davis et al., 2009; Carpenter et al., 2013). An inverse relationship between obesity and D2R availability, assayed via positron emission tomography (PET), has also been reported in humans. This was first reported by Wang et al. (2001) and was initially supported by others (Volkow et al., 2008; de Weijer et al., 2011; Kessler et al., 2014; van de Giessen et al., 2014). However, several other groups have failed to replicate this finding (Dunn et al., 2012; Caravaggio et al., 2015; Cosgrove et al., 2015; Karlsson et al., 2015, 2016; Tuominen et al., 2015), or found opposing associations in different regions of the striatum (Guo et al., 2014). Interestingly, Guo and colleagues noted a negative relationship between body mass index (BMI) and D2R binding only in the ventral striatum, which may be linked to effortful movements (Salamone et al., 2007; Schmidt et al., 2012). Several possibilities may account for the discrepancy among studies of D2R binding and BMI. Different D2R radio-ligands were used among these studies, which may bind differentially to D2R or D3Rs (Gaiser et al., 2016). Changes in striatal dopamine tone could impact binding potential (Horstmann et al., 2015). Finally, experimental factors including the amount of time after meal consumption or individual variability among subjects may contribute to observed differences (Small et al., 2003).

Animal studies have more consistently linked impairments in D2Rs to obesity, via analysis of mRNA (Mathes et al., 2010; Zhang et al., 2015), protein (Johnson and Kenny, 2010; Adams et al., 2015), and receptor binding (Huang et al., 2006; Hajnal et al., 2008; Thanos et al., 2008; Michaelides et al., 2012; van de Giessen et al., 2012, 2013; Narayanaswami et al., 2013). Interestingly, rats maintained on an iso-caloric high-fat (but not high-sugar) diet also had lower levels of D2Rs in ventral (but not dorsal) striatum (Adams et al., 2015), supporting the conclusion that exposure to high-fat diet may be a better predictor of dopaminergic dysfunction than weight gain itself (van de Giessen et al., 2013). To date, no published work has examined associations between D1-type dopamine receptors (D1Rs) and obesity in humans, so an evaluation of potential changes here is limited to a small number of animal studies. D1R mRNA was decreased in obese rats relative to lean controls (Vucetic et al., 2012; Zhang et al., 2015), while another study reported a decrease in D1Rs only in female rats (Ong et al., 2013). We conclude that reduced function of D2Rs appears to be a particularly important alteration in obesity, although there is considerable variability in D2R alterations among studies and individuals. Unfortunately, studies of the D1R are too sparse to make strong conclusions about its relationship to obesity.

## Do alterations in dopamine function recover with weight loss?

It is unclear whether changes in dopamine signaling in people with obesity persist after weight loss. The few studies that exist on this topic point to dopaminergic alterations being at least partly resistant to change, and at times even exacerbated by weight loss. High-fat diet reduced the levels of several enzymes involved in dopamine production in the VTA and NAc, and switching these obese mice to low-fat chow caused even further decreases in these enzymes (Carlin et al., 2013; Sharma et al., 2013). Two PET imaging studies reported a lack of recovery of D2R binding following Roux-en-Y gastric bypass surgery (RYGB) in humans, with one showing an even further decrease in binding (Dunn et al., 2010; de Weijer et al., 2014). A small study of five women reported a partial recovery of D2R binding 6-weeks after RYGB (Steele et al., 2010). An increase in D2R binding was also reported during food restriction and associated weight alterations in obese rats (Thanos et al., 2008). Although the data on this topic are limited, it appears that diet-induced changes in dopamine function are at least partly persistent following weight loss. Consistent with this conclusion, physical activity levels remain low in people with obesity, even months after the peak of weight loss (Bond et al., 2010; Camps et al., 2013; Ramirez-Marrero et al., 2014; Berglind et al., 2015, 2016). Again, the small number of studies of this topic precludes firm conclusions, and underscores the need for further research on the persistence of dopaminergic alterations in people with obesity.

## Obesity and physical inactivity: conclusions

Chronic exposure to obesogenic diets is associated with changes in both physical activity levels and dopaminergic function. Diet-induced changes in the dopamine system may be sufficient to explain the development of physical inactivity in people with obesity. Increased understanding of obesity-related changes in dopamine and related systems may support evidence-based approaches for increasing physical activity in people with obesity. In addition, such an understanding may reveal genetic or environmental contributions to dopaminergic dysfunction, and physical inactivity, in obesity.

## Author contributions

AK, TO, and DF conceived of the idea and wrote and edited this manuscript.

### Conflict of interest statement

The authors declare that the research was conducted in the absence of any commercial or financial relationships that could be construed as a potential conflict of interest.
